# Vivaldi Antennas for Contactless Sensing of Implant Deflections and Stiffness for Orthopaedic Applications

**DOI:** 10.1109/access.2021.3137718

**Published:** 2021-12-23

**Authors:** JAKOB G. WOLYNSKI, MILAN M. ILIĆ, BRANISLAV M. NOTAROŠ, KEVIN M. LABUS, CHRISTIAN M. PUTTLITZ, KIRK C. MCGILVRAY

**Affiliations:** 1 Department of Mechanical Engineering, Colorado State University, Fort Collins, CO 80523, USA; 2 School of Electrical Engineering, University of Belgrade, 11120 Belgrade, Serbia; 3 Department of Electrical and Computer Engineering, Colorado State University, Fort Collins, CO 80523, USA

**Keywords:** Antenna for orthopaedic application, antenna resonant frequency, computational electromagnetic simulations, electromagnetic sensor, external sensing of object displacement/deflection/stiffness, monitoring and predicting bone fracture healing, near electromagnetic field, resonant frequency measurements, Vivaldi antenna

## Abstract

The implementation of novel coaxial dipole antennas has been shown to be a satisfactory diagnostic platform for the prediction of orthopaedic bone fracture healing outcomes. These techniques require mechanical deflection of implanted metallic hardware (i.e., rods and plates), which, when loaded, produce measurable changes in the resonant frequency of the adjacent antenna. Despite promising initial results, the coiled coaxial antenna design is limited by large antenna sizes and nonlinearity in the resonant frequency data. The purpose of this study was to develop two Vivaldi antennas (a.k.a., “standard” and “miniaturized”) to address these challenges. Antenna behaviors were first computationally modeled prior to prototype fabrication. In subsequent benchtop tests, metallic plate segments were displaced from the prototype antennas via precision linear actuator while measuring resultant change in resonant frequency. Close agreement was observed between computational and benchtop results, where antennas were highly sensitive to small displacements of the metallic hardware, with sensitivity decreasing nonlinearly with increasing distance. Greater sensitivity was observed for the miniaturized design for both stainless steel and titanium implants. Additionally, these data demonstrated that by taking resonant frequency data during implant displacement and then again during antenna displacement from the same sample, via linear actuators, that “antenna calibration procedures” could be used to enable a clinically relevant quantification of fracture stiffness from the raw resonant frequency data. These improvements mitigate diagnostic challenges associated with nonlinear resonant frequency response seen in previous antenna designs.

## I. INTRODUCTION

Orthopaedic bone fracture healing complications remain pervasive, despite ongoing improvements to clinical standards of care. Long bone fractures, such as those of the tibia, exhibit failed healing (nonunion) in up to 12% of cases [1]. Fracture nonunions are extraordinarily harmful to patient wellbeing, ultimately leading to the need for additional surgical intervention, chronic pain, increased opioid usage, and an estimated 118% increase in medical expenditures [1], [2]. Healing outcome can be improved, and patient suffering reduced, in cases where rapid diagnosis of adverse fracture healing is achieved to advise corrective therapies during the early stages (< 30 days post-surgery) of fracture healing [3], [4].

Achieving an early prediction of healing outcome remains difficult using current clinical diagnostic tools: namely, biplanar x-ray imaging. This qualitative and subjective technique has proven to lack specificity [5]–[8] which limits the reliability of x-ray image predictions of healing outcome [9], and further increases mean nonunion diagnosis times to exceed 6 months [10]. The clinical deficit resulting from the imperative need for rapid prediction of adverse fracture healing, coupled with the apparent inefficacy of x-ray imaging techniques, necessitates the development of new orthopaedic diagnostic techniques.

Ongoing efforts have pursued development of technologies to quantify *in vivo* fracture biomechanics [11], [12], as fracture stiffness has demonstrated reliability as an early predictor of healing [12]–[19]. These techniques exploit differences in the temporal progression of fractures trending towards proper or adverse healing outcomes. Proper bone fracture healing causes a progressive increase in fracture tissue (callus) stiffness; conversely, adverse healing presents temporal invariance in callus stiffness progression [20]. These mechanical patterns are apparent during early phases of fracture healing, prior to x-ray appearance of radiopaque tissues, thus biomechanical methods exhibit promise as means to expedite accurate prediction of healing outcome [18], [21].

Bone fractures are ubiquitously treated by implantation of metallic orthopaedic hardware (i.e., rods, plates, and screws) to stabilize and support bone fragments. The bone-implant behaves as a composite structure whose biomechanical properties reflect contributions from both members; thus, temporal changes to the callus are apparent in the stiffness changes of the composite. Previous efforts have been made to leverage this phenomenon for diagnostic purposes, by instrumenting orthopaedic hardware with sensors to telemetrically report the hardware’s mechanical environment (i.e., strain or load share) [20]–[23]. Despite promising results, this technique typically requires hardware modification to accommodate sensor architecture, which precludes use with existing orthopaedic hardware types and may contribute to premature/traumatic implant failure [24].

To address these challenges, a diagnostic antenna system was developed for noninvasive quantification of relative changes in fracture stiffness for any case treated with off-the-shelf metallic orthopaedic hardware [25]–[27]. This diagnostic device utilized a coiled coaxial dipole antenna design to directly electromagnetically couple (DEC) to metals in its near-field, thus enabling detection of displacements of metallic implant hardware [25]. By applying controlled physiologically non-detrimental mechanical loads to the fractured limb, implanted metallic hardware could be accurately displaced towards the DEC antenna. Resultant deflections produced a measureable change in the resonant frequency of the antenna, where the magnitude of implant deflections were a function of the fracture stiffness, a direct measure of healing. This technology has exhibited aptitude in quantifying relative changes in healing fracture stiffness in translational cadaveric [26] and *in vivo* fracture models [27]. However, this antenna’s predictions of implant displacements are highly sensitive to the initial antenna-implant distance which can be neither known nor controlled with confidence in a clinical setting. Specifically, imperceptibly small variations in antenna placement across temporally repeated diagnostic measurements consequently produce sufficient variance to obfuscate healing progression patterns.

We hypothesize that the ability to perform antenna calibrations will surmount these limitations by enabling accurate conversion of resonant frequency shift into implant displacements. However, these calibrations require the ability to precisely displace the antenna while performing diagnostic DEC measurements, thus quantifying resonant frequency shifts for a known change in antenna implant distance. The large size of the previous coaxial cable antenna design makes this infeasible, hence there is an imperative need for DEC antennas with an appreciable size reduction. Within this study, the applicability of Vivaldi-type antennas for this task was explored; to our knowledge, this has not been investigated nor exploited elsewhere.

Vivaldi-type antennas are known to have excellent broadband characteristics, low cross polarization, and directive radiation patterns [28]. They are widely used in broadband applications [29], and have recently been used as contactless sensors [30]. A Vivaldi-type antenna was primarily chosen for the considered application due to its simple design, low fabrication cost, ease of fabrication on a printed circuit board (PCB), simple feeding network providing internal matching with no need for tuning, and a thin profile. The thin profile of this antenna design facilitates mounting on any positioning rig, as well as adding additional elements and forming an array for simultaneous multi-element sensing, which is ideal for orthopaedic applications.

The objective of this study was to explore the sensitivity of Vivaldi antennas for detecting movement of metal plates in the near field. Standard and miniaturized antenna designs were evaluated by computational modelling to predict antenna sensitivity to changes in metallic plate displacements; analogous physical experiments were performed using fabricated prototype antennas. Physical experiments were parametrically varied to evaluate the effects on antenna sensitivity related to alloy of the metallic plate, antenna orientation relative to the metallic plate, and whether antennas could be spatially adjusted following data collection to facilitate antenna calibration. The resultant data were used to inform the feasibility to utilize Vivaldi antennas for applications as orthopaedic diagnostic sensors.

## II. METHODS

### A. VIVALDI ANTENNA DESIGN

**FIGURE 1.**
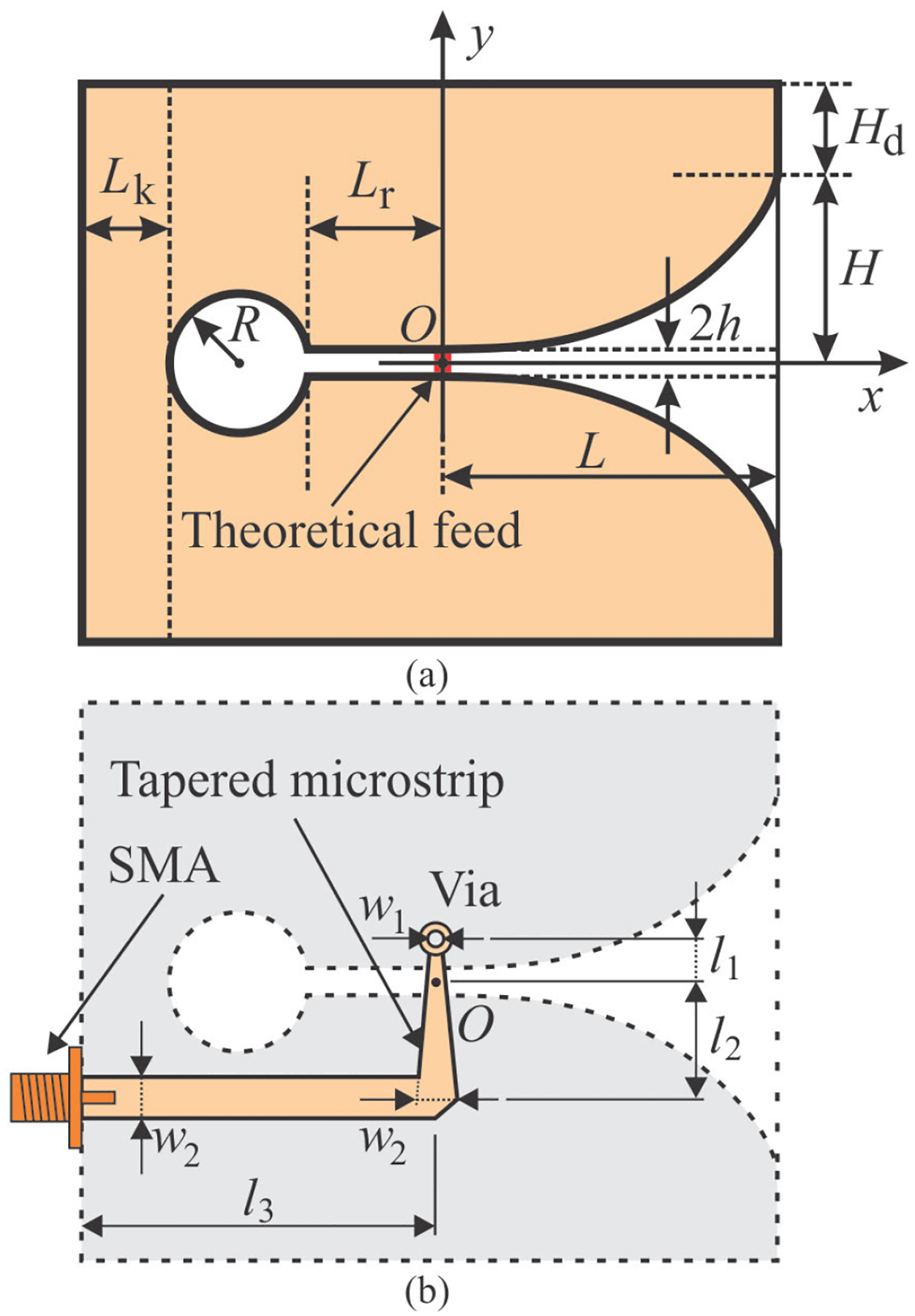
A sketch of a planar Vivaldi antenna with relevant geometrical parameters: (a) top side of a PCB with theoretical point-like feed and (b) bottom side with actual feeding network.

Vivaldi antennas belong to a class of tapered slot antennas. The antenna operation is most easily understood if the planar copper structure (Fig. 1a) is imagined as an axial cross section of a body of rotation (BoR), obtained by rotating the cross section around the *x*-axis, since it resembles a cavity-backed open ended horn. With this in mind, one can easily understand the expectation of the antenna to be highly sensitive to frontally positioned structures, which is a main desired feature in the intended orthopedic application.

Planar Vivaldi antennas [31], manufactured on PCB, were considered as the basic sensing element for the intended application. The top side (Fig. 1a) is comprised of a copper plating residing on a thin dielectric substrate (i.e., essentially forming an open ended slot line). Fig. 1a also shows a theoretical feed, i.e., a lumped point-source generator connected between the top and bottom metallic slot liners. This feed was used only in initial assessment of the antenna performance, without the dielectric substrate, because it facilitated rapid simulations which were mandatory for multiple parametric sweeps and enabled near real-time tuning. On the bottom side (Fig. 1b) is an actual feeding network comprised of a simple tapered microstrip line acting as an impedance transformer, metalized through via, and subminiature version A (SMA) PCB-mount connector which facilitated straightforward link to a vector network analyzer (VNA) by coaxial line.

The symmetric slot-like section in the antenna middle (Fig. 1a) was characterized [31] by a circular section (i.e., a short ended slot line of radius *R*) followed by a straight slot of length *L*_r_ and half-width *h*, followed by a slot of length *L*, and exponentially tapered half-width ranging from *h* to *H*, whose tapering edge was governed by the following equations, where *a* is a normalizing parameter:

(1) $$y=Ae^{ax}+B$$

(2) B=h−A,

(3) $$A=\frac{H-h}{{\text{e}}^{aL}-1}$$

The copper plating extends to the left of the circle and, symmetrically to the top and bottom of the tapered slot by lengths *L*_*k*_ and *H*_*d*_, respectively. The feeding point (i.e., via) was located at *x* = 0, its vertical position was very close to the conductor, edge with slightly adjustable *y*-axis position to facilitate impedance matching (Fig. 1b). The width and length of the tapered microstrip feeding line were obtained by optimizing the impedance matching of the antenna to the standard 50-ohms.

When measuring through biological tissues, increasing electromagnetic frequency results in greater attenuation [32], [33]. However, higher frequency bands, i.e., around 1.5 GHz, enable larger absolute shifts in resonance of the antennas with obstacles positioned in the near field, while still allowing sufficient tissue penetration. Hence, operation at frequencies around 1.5 GHz (1.2 GHz and slightly above) was targeted. The antenna, termed as “standard”, was designed for distinct variation of resonances about this frequency with respect to change in position of front-mounted metallic structures. At the same time, broadband characteristics of the “standard” antenna allowed assessment of antenna performance at higher bands. Successful detection of metallic plate position in preliminary simulations near 1.2 GHz, which included bone, muscle, and skin, validated the proposed design practicality (with proper calibration), and also warranted miniaturization of the “standard” antenna by making it significantly smaller, and unavoidably narrow band, while preserving its sensitivity to front-mounted obstacles. Minimal antenna size was an important design consideration for the intended use as an orthopaedic sensor, as well as for its future integration in a small array for increased sensitivity and resolution; thus, a “miniaturized” version of the Vivaldi antenna was analyzed, which was similarly optimized with respect to position of front-mounted metallic structures. In the miniaturization process, the initial parameters, *L* and *H*, were systematically made smaller, after which the remaining parameters were reoptimized to yield good sensitivity at approximately 1.5 GHz. Finally, the real feed was adjusted to accommodate the changes in dimensions and provide good 50-ohm matching. The “miniaturized” antenna design parameters are given in Table 1.

### B. ANTENNA SIMULATION

**FIGURE 2.**
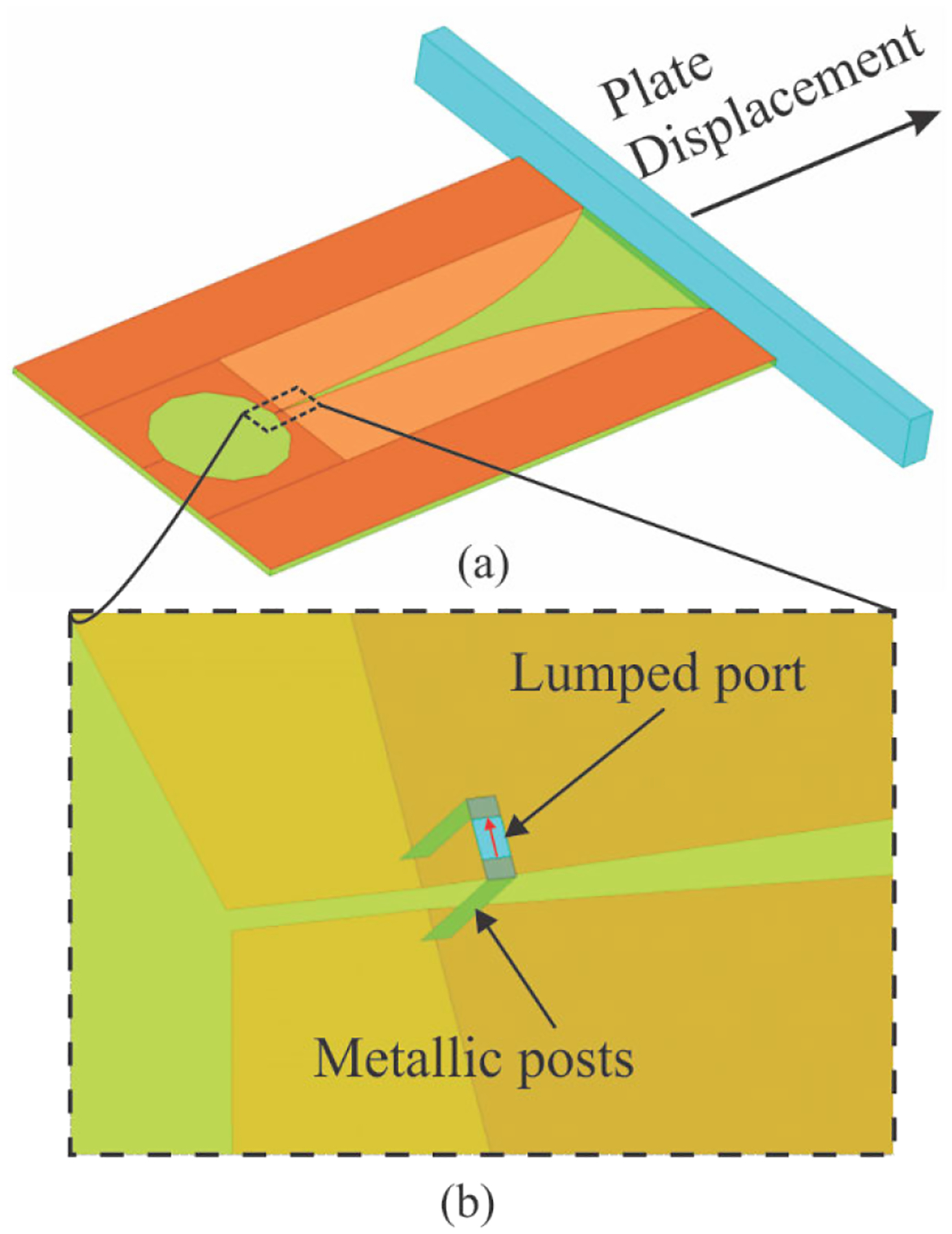
A full-wave EM model of the Vivaldi antenna with geometrical parameters from Table 1. (a) Top side of the antenna with metallic plate placed in front of its measurement side. (b) A magnified view of the simple lumped-port feed attached at the bottom of the substrate.

For the purpose of computer simulation (*in silico*) of antenna performance prior to prototype production, each antenna was modeled in a full-wave three dimensional (3-D) electromagnetic (EM) simulator (ANSYS HFSS, ANSYS; Canonsburg, PA) (Fig. 2). As the next step in the progression of increasing the feed and model complexities, i.e., after employing the theoretical feed and prior to design of the real feed shown in Fig. 1b, this model employed an actual dielectric substrate (discussed in section C), a simple bridge feed, and a lumped generator port. The feed is comprised of two metallic strip posts running vertically through the dielectric and a port residing on the dielectric surface (highlighted in cyan in Fig. 2b). Metallic surfaces of the antenna were modeled as infinitely thin sheets. The primary interest was the antenna near field characteristics, thus far field parameters were not investigated. The model was encased in an air box and absorbing boundary condition (ABC) was applied at its faces to truncate the numeric domain. Full-wave simulations were carried out at a reference frequency of 2 GHz to ensure optimal convergence at both lower and slightly higher frequencies. An initially seeded small domain tetrahedral mesh and first-order basis functions for field expansion were used. Near monotonic convergence to a maximal magnitude of *S*-parameter variation lower than 0.002 was typically achieved within 10 adaptive passes employing approximately 33000 elements, 209000 unknowns. Antenna reflection coefficient (*S*_11_) was simulated over a range of frequencies (0.3 – 3.0 GHz) using an interpolating sweep.

A metallic obstacle (i.e., a plate) was modeled as a perfect electric conductor (PEC) (dimensions of 152 × 12 × 6 mm), and was symmetrically positioned in front of the antenna (Fig. 2a). For both antenna designs, *S*_11_ was simulated over a frequency broad band (0 – 6 GHz). Simulations were repeated for increasing distance / offset between the plate and antenna (0.1, 0.5, 1, 2, 3, 4, 6, 8, and 10 mm). Antenna resonance near 1.5 GHz was predicted for each plate offset.

### C. PROTOTYPE ANTENNA PRODUCTION

**FIGURE 3.**
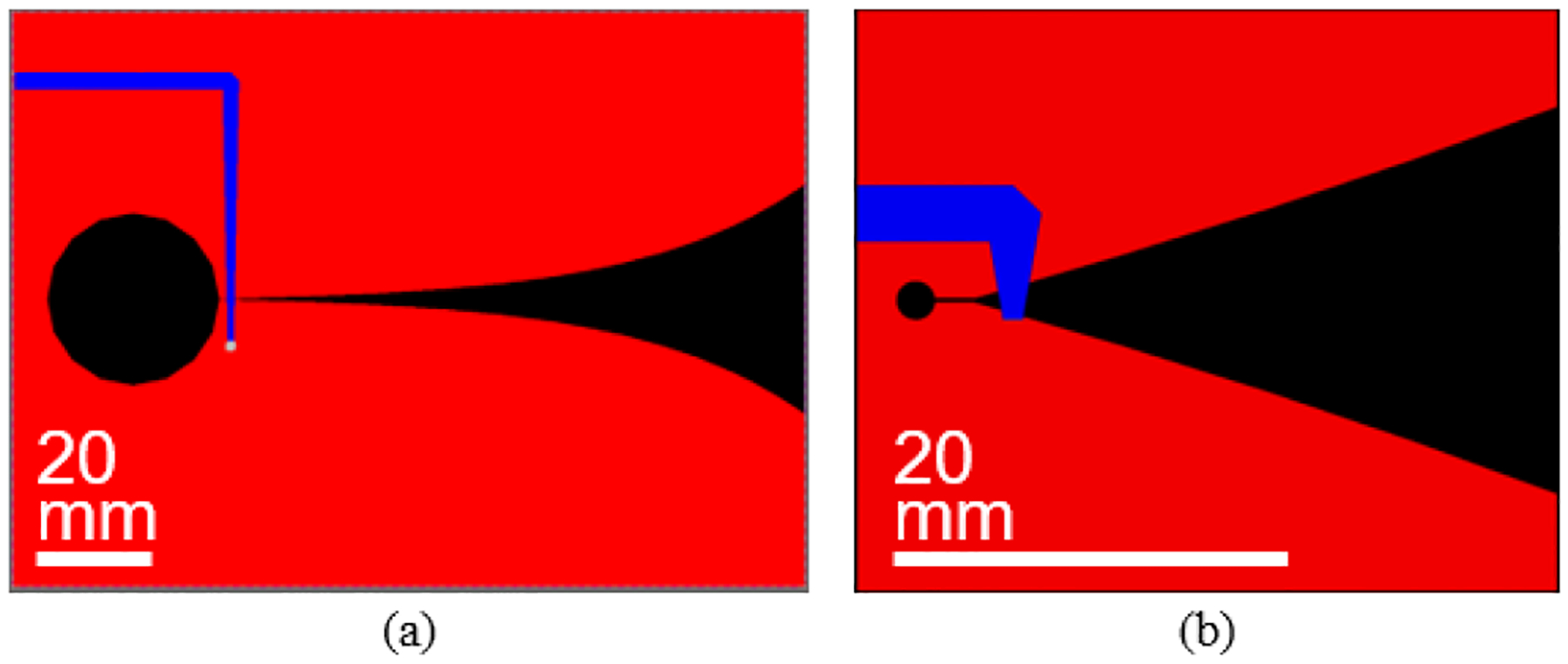
Layout of the (a) standard and (b) miniaturized Vivaldi antenna designs, showing top copper layer in red and bottom layer (feeding strip) in blue. Feature dimensions within each layout are proportionately accurate, but scaling of each layout are different to improve visibility (accurate dimensions for each layout are detailed in Table 1).

Following *in silico* characterizations of the antennas, prototypes were fabricated for benchtop testing validation. The feeding microstrip line for both Vivaldi antenna designs were optimized to obtain good impedance matching to 50-ohms (Fig. 3). Note that differently optimized and more complex feeding networks could have been implemented, including those with broadband radial stubs [29]. However, the presented microstrip lines with simple through-via connections proved sufficient for the targeted application. Our design opted for a *h*_*s*_ = 1.57 mm thick FR-4 substrate (*ϵ*_*r*_ = 4.4, tan*δ* = 0.02) with *t*_*s*_ = 35 *μ*m thick double sided copper metallization. Note that these materials were employed with the exact parameters from the simulation models. The only exception was the metallization thickness, which was neglected in simulations without loss of accuracy at the considered frequencies. The layouts of the optimized antenna designs were transferred from the EM simulator to a PCB design tool, KiCad, for requisite file generation. Final antennas were manufactured using photolithography and chemical etching. Since the PCB production allowed automatic via metallization, the only additional antenna assembly process was the soldering of the SMA connector. No additional matching networks or tuning was required because the antennas were internally matched.

### D. PROTOTYPE ANTENNA SENSITIVITY TO METALLIC STRUCTURE DISPLACEMENTS

**FIGURE 4.**
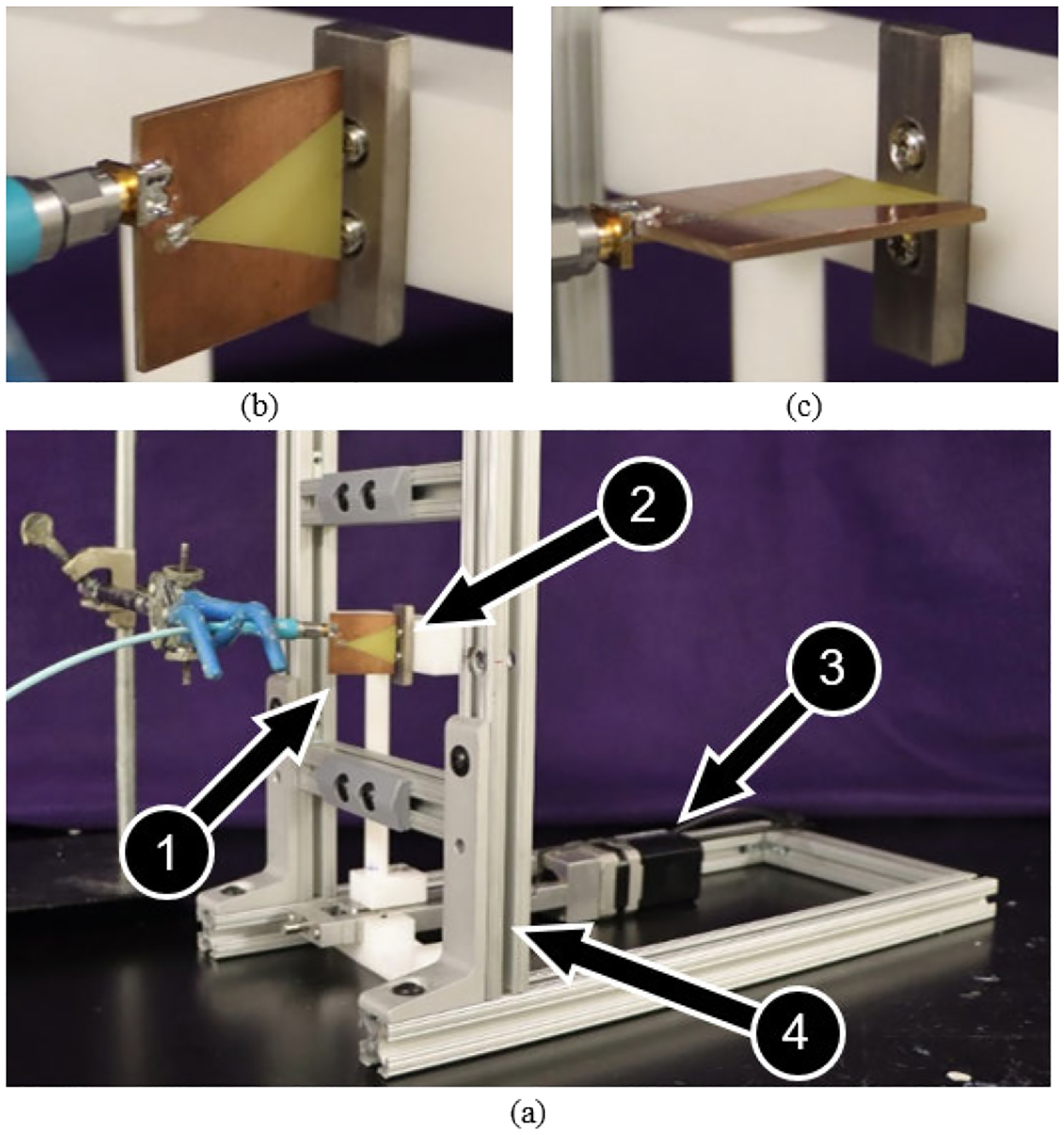
(a) The prototype antenna (1) was positioned such that it was aligned either (b) parallel or (c) perpendicular to a metallic plate. The plate was attached to a precision linear actuator (3) so that antenna-plate displacement could be precisely increased while measuring the resultant shift in resonant frequency. The antenna was surrounded by an aluminum frame (4) to recapitulate the testing environment necessary for use as an orthopaedic diagnostic device.

To evaluate the sensitivity of the prototype antennas to changes in antenna-implant distance, a stainless steel (SS) plate (40 mm × 20 mm × 10 mm) was secured to a precision linear actuator (T-LLS105; Zaber Technologies; Vancouver, BC, Canada; 0.15625 *μ*m microstep resolution) so that change in resonant frequency (resonant frequency shift) could be measured while progressively increasing the antenna-plate distance (Fig. 4). To mitigate potential off-target coupling, the plate was offset from the actuator using non-conductive nylon arms, where the plate was secured to the nylon using orthopaedic screws (3.5 mm diameter, 316L stainless steel). To recapitulate the testing environment relevant to how the antenna would be used as an orthopaedic diagnostic tool, the antenna was positioned within a surrounding aluminum construct (150 × 150 mm internal frame dimensions) (Fig. 4).

The starting position of the plate was in direct contact with the measuring edge of the antenna, and was displaced from the antenna (0 – 10 mm, 0.01 mm increments, n ≈ 5 data points collected at each position) while antenna resonant frequency shifts were measured. Resonant frequency was determined as the frequency at which minimum *S*_11_ occurred, as measured by a VNA (TTR500; Tektronix; Beaverton, OR; 200 MHz span linearly distributed over 500 points, 7 dBm power). Antenna sensitivity was calculated by taking the slope of a linear fit applied to a 0.5 mm window of resonant frequency shift-displacement data. These methods were repeated for parametric variations of prototype antenna design and antenna orientation (i.e., antenna and plate lengths being parallel or perpendicular, Fig. 4). The miniaturized antenna was designed with intended biomedical applications, and thus antenna sensitivity to orthopaedically relevant metallic alloys was a primary concern. Accordingly, tests for the miniaturized antenna design included an additional parameter: use of SS or titanium (Ti) plate materials (dimensionally equivalent plate designs).

An additional test was performed using the miniaturized antenna, positioned perpendicular to a SS plate segment (Fig. 4c). In this test, the antenna and plate segment were connected to separate precision linear actuators, with the movement direction of each actuator being collinear. An initial test was performed in which resonant frequency shifts were collected while displacing the plate, following the methods of the preceding paragraph. Upon test completion, the plate was returned to its initial location, and the test was repeated while instead displacing the antenna. The purpose of this study was to evaluate the hypothesis that resonant frequency shifts were repeatable for any antenna-plate displacement scenario, regardless of which of the two members were displaced. Despite the simplicity of this test, the findings were of paramount interest for orthopaedic applications, as discussed later.

## III. RESULTS

### A. ANTENNA SIMULATION

**FIGURE 5.**
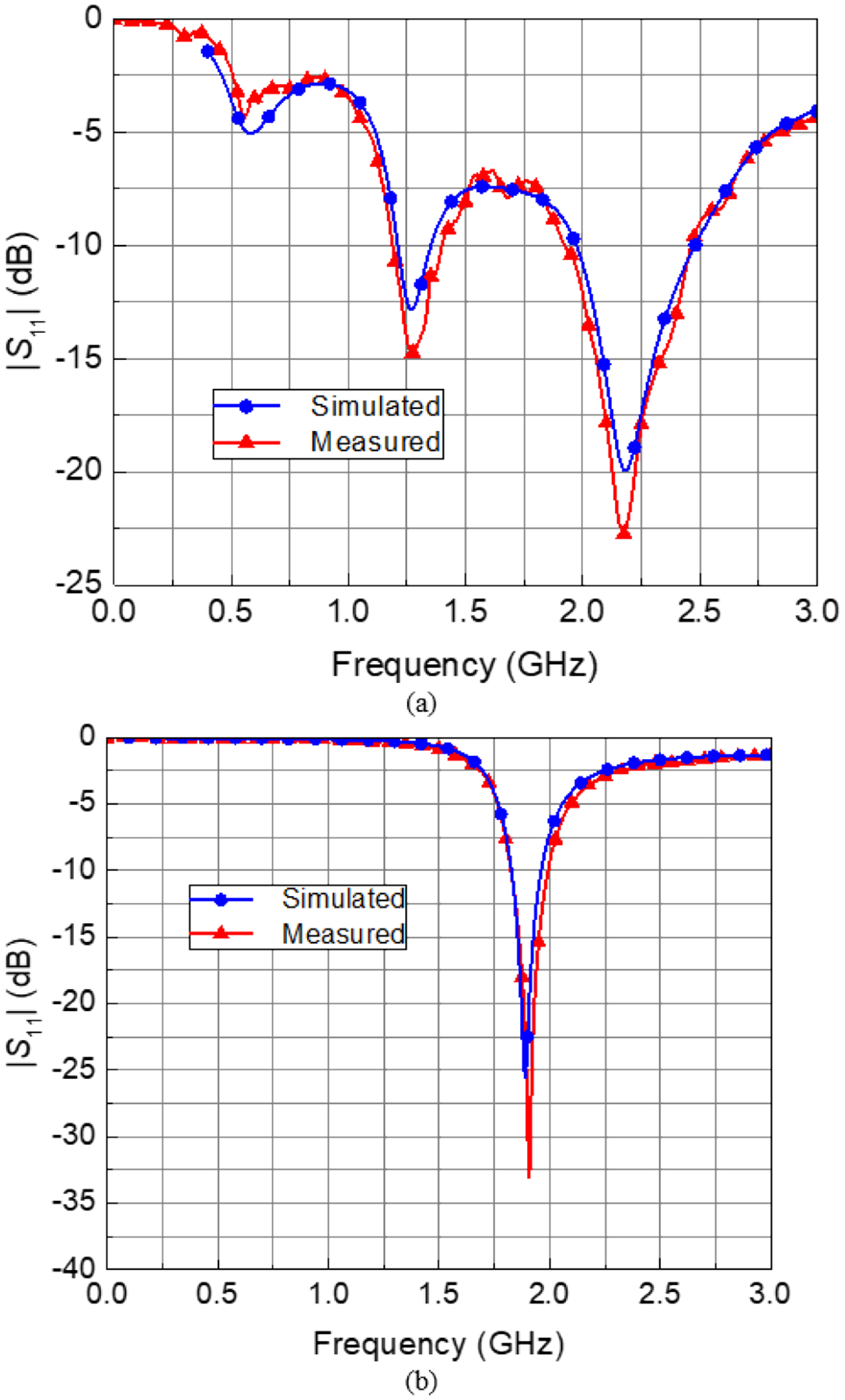
Comparison of simulated and prototype measurements of antenna *S*_11_ versus frequency for the (a) standard and (b) miniaturized antenna designs.

Fig. 5 shows a comparison of antenna *S*_11_ obtained by simulations and by measurements on a fabricated prototype, in free space, using a calibrated VNA.

**FIGURE 6.**
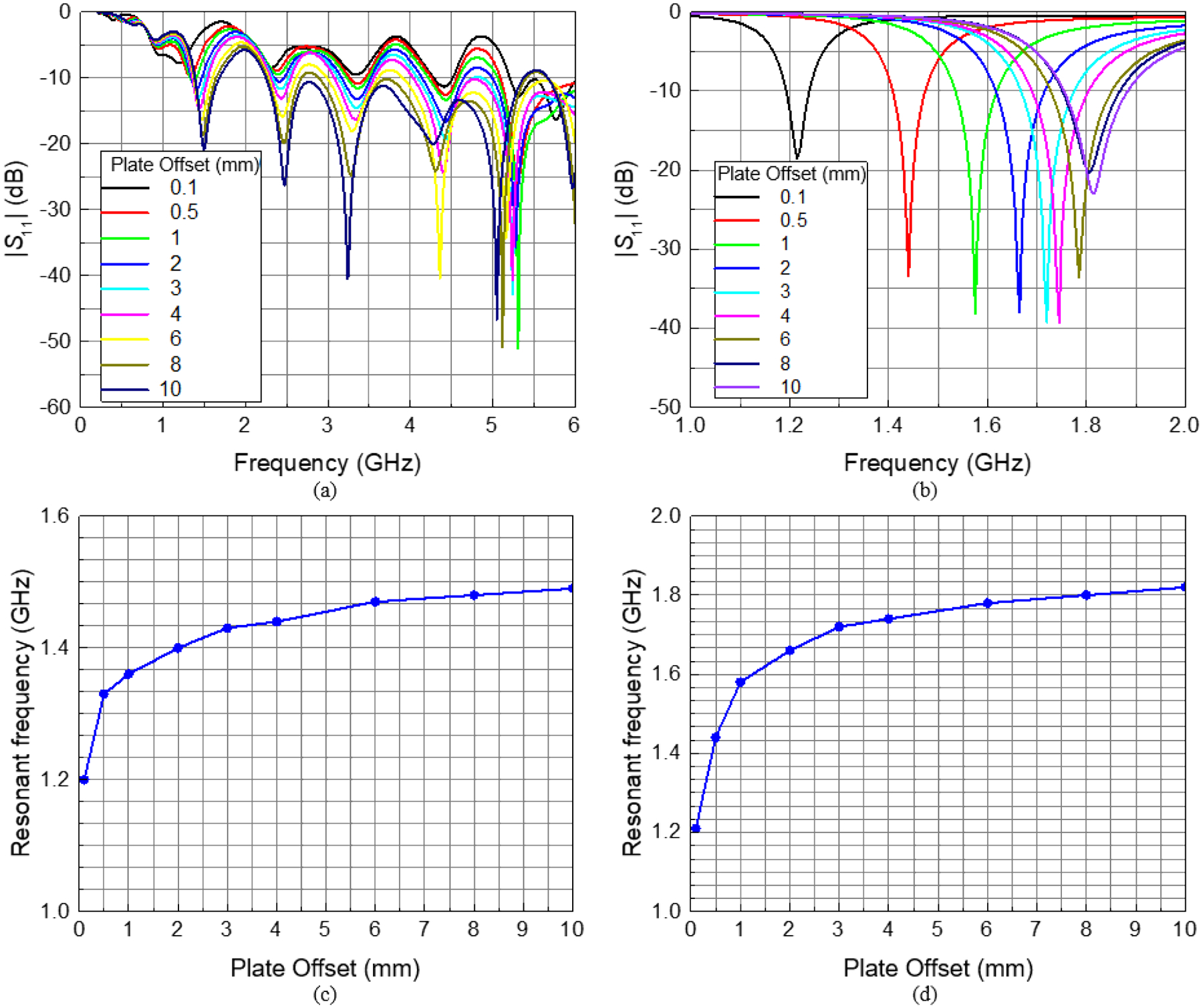
Simulated measurements of antenna *S*_11_ versus frequency for the (a) standard and (b) miniaturized antenna designs, as a function of metallic plate offset from the antenna. Resonant frequency (resonance near 1.5 GHz) for the (c) standard and (d) miniaturized antennas.

Fig. 6a–b shows simulated predictions of *S*_11_ behavior for broadband (up to 6 GHz) in the standard antenna, which was discussed in Section IIA, and near the operational frequency of interest (1.5 GHz) for the miniaturized antenna. A family of curves in the figure was obtained for various metallic plate offsets (i.e., distances from the front of the antenna). For the standard antenna, resonances were predicted around 1.5 GHz, 2.5 GHz, 3.2 GHz, 4.5 GHz and above.

For the resonance of interest (near 1.5 GHz), resonant frequency increased non-linearly with increasing antenna-metallic plate distance (Fig. 6c–d, Table 2). For both the standard and miniaturized antennas, predicted resonant frequency increases resulting from the first 1 mm of metallic plate offset (0.16 and 0.37 GHz, respectively) were greater than those for the next 9 mm of offset (0.13 and 0.24 GHz, respectively; Table 2).

### B. PROTOTYPE ANTENNA SENSITIVITY TO METALLIC STRUCTURE DISPLACEMENTS

**FIGURE 7.**
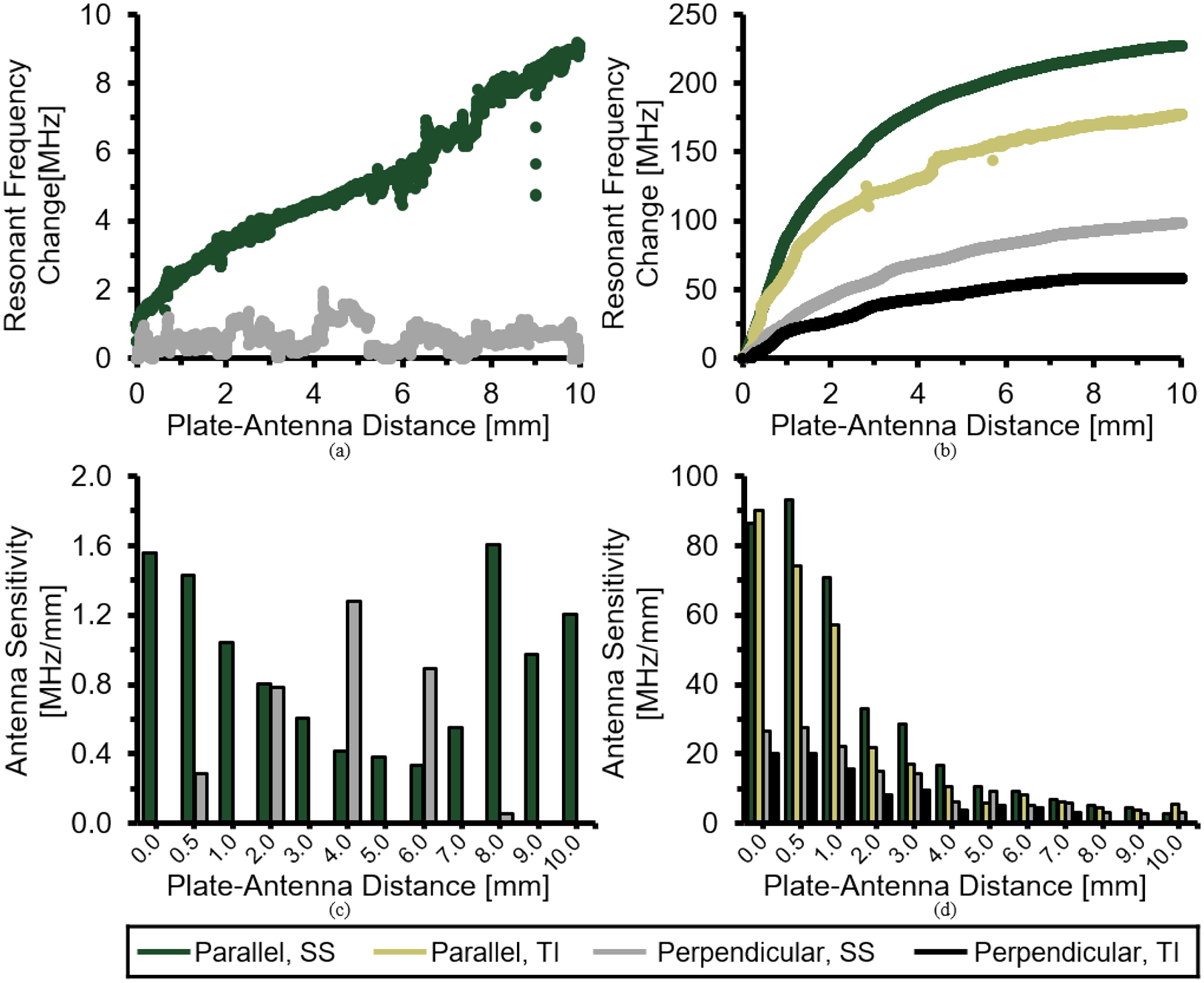
Change in resonant frequency measured while increasing distance between (a) standard or (b) miniaturized antenna and metal plate segment of SS or Ti alloy. Tests were repeated with the antenna aligned parallel or perpendicular to the plate segment (Fig. 4). Antenna sensitivity of the (c) standard or (d) miniaturized antennas were calculated by taking the slope of a linear fit applied to a 0.5 mm window of resonant frequency shift-displacement data in (a) or (b), respectively.

During physical tests, antenna resonant frequency increases were observed as the distance between antenna and SS plate segments were increased via linear actuator (Fig. 7a–b); however, the magnitude of these increases were smallest for the standard antenna, and clear data trends were absent when this antenna was positioned perpendicular to the metal plate (Fig. 7a).

As distance was increased from 0 – 10 mm, resonant frequency increases of 227.2 MHz and 98.5 MHz were observed for tests in which the miniaturized antenna was oriented parallel or perpendicular to a SS plate segment, respectively (Fig. 7b). Similar analysis for a Ti plate segment yielded total resonant frequency increases of 177.7 and 58.1 MHz, respectively. For all miniaturized antenna tests, sensitivity (i.e., the instantaneous slopes at various displacement values of Fig. 7a–b) was largest for plate-antenna distances of less than 1 mm, and tended to non-linearly decrease with increasing plate-antenna distance. For distances less than 10 mm, antenna sensitivities were larger when the miniaturized antenna was positioned parallel, instead of perpendicular, to the plate segment (Fig. 7d).

**FIGURE 8.**
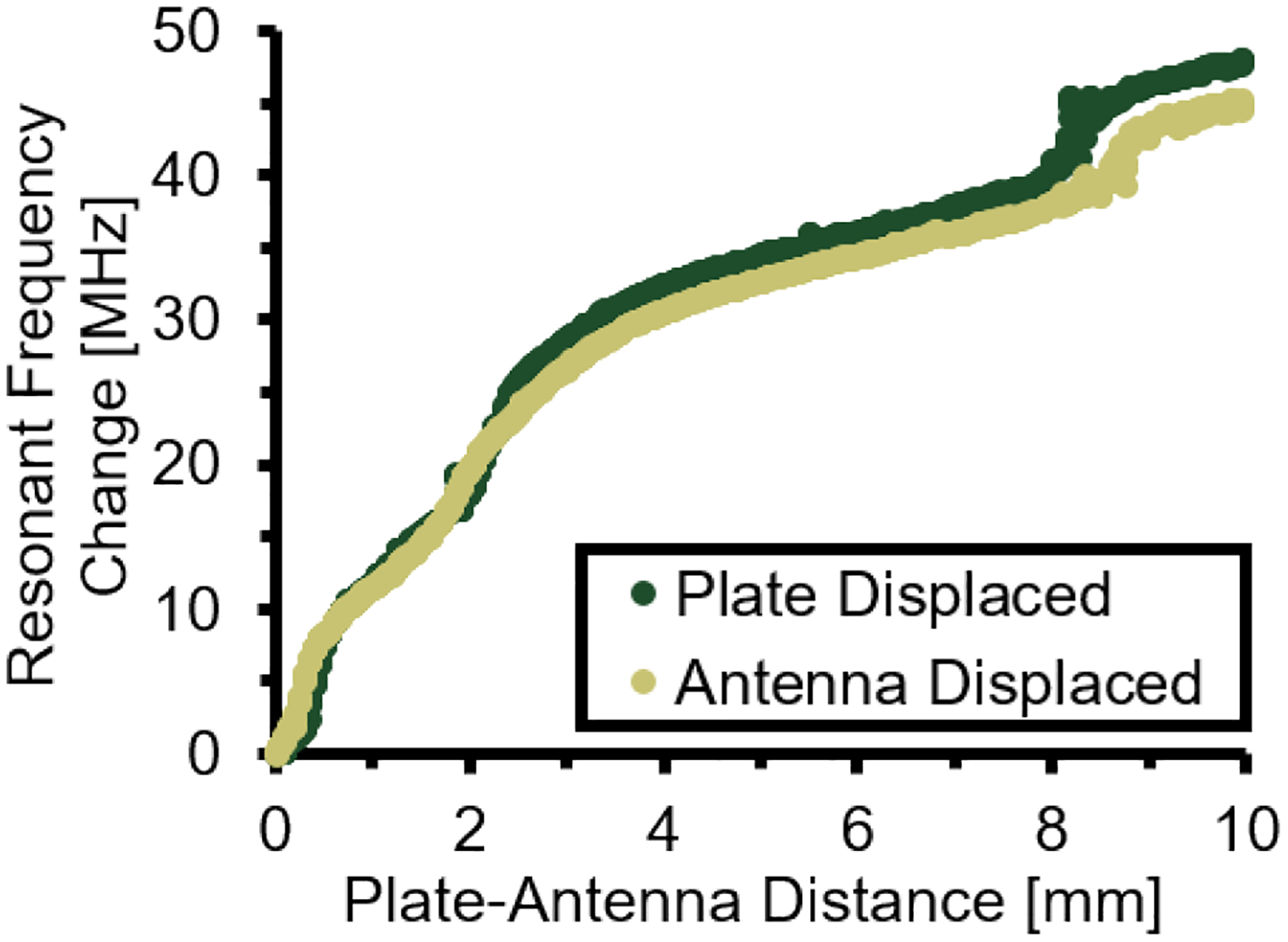
Resonant frequency shifts associated with the miniaturized antenna positioned perpendicular to a SS plate segment. Plate-antenna distance was increased by linear actuator displacement of either the plate (antenna remained stationary) or antenna (plate remained stationary).

Resonant frequency shifts were similar for tests in which the plate-antenna distance was increased by displacing the plate or displacing the antenna (Fig. 8). For the first 0.5 mm of displacement, resonant frequency shifts tended to be larger for the test in which the antenna was displaced, and the average difference in resonant frequency shift for any given plate-antenna distance in this range was 1.7 MHz. For all plate-antenna distances of 0.5 mm or larger, resonant frequency shifts for each method averaged 5.2% difference, and did not exceed 10% difference.

## IV. DISCUSSION

*In silico* simulations of the two proposed Vivaldi antenna designs established the efficacy of the antennas, with regards to their intended application of sensing metallic orthopaedic implant deflections, prior to fabrication. The validity of these simulations was supported by the apparent agreement in simulated and prototype measurements of antenna *S*_11_ versus frequency data for both antenna designs (Fig. 5).

Computational predictions of antenna behavior, in the presence of a metallic plate, further indicate that standard antenna resonances can be observed around 1.5 GHz, 2.5 GHz, 3.2 GHz, 4.5 GHz and above. Out of these sets, the resonances around 1.5 GHz (as well as around 3.2 GHz and possibly others) are particularly favorable due to their easily distinguishable resonant frequencies, which are well separated for different plate distances (Fig. 6a). The data of Table 2 support the conclusion that resonances are pronounced, sharp, well separated, and easily distinguishable by both frequency and magnitude.

The validity of these computational simulations are further bolstered by the similarity in predicted miniaturized antenna resonant frequency shift (Fig. 6d), to the data obtained from comparable physical experiments with a prototype antenna (Fig. 7b). Experimental agreement was worse for the standard antenna design (Fig. 6c and Fig. 7a), although this can ostensibly be attributed to the difference in metallic plate dimensions for the two methodologies. The computational experiments modeled a large plate segment which covered the entire antenna opening, while benchtop experiments used a smaller plate segment (intended to recapitulate the region of interest for orthopaedic applications) which covered only 20% - 40% of the measurement side of the antenna (perpendicular and parallel orientations, respectively).

We can conclude from the miniaturized antenna data sets that resonant frequency shifts are highly sensitive at small distances between a metallic object and the antenna; these sensitivities become increasingly diminished as the plate offset increases (Fig. 7d). Benchtop results further exhibit the efficacy of the Vivaldi antennas to detect changes in antenna-plate distance for a variety of metallic alloys relevant to orthopaedic applications (i.e., stainless steel and titanium). These data, however, advise that antenna sensitivities tend to be slightly elevated for stainless steel relative to titanium, and further suggest that sensitivity was maximized when the metallic structure covered greater portions of the measurement edge of the antenna (i.e., antenna parallel to implant). Based on these findings, it can be concluded that the miniaturized antenna is well suited to detect relative displacements of any metallic orthopaedic structure, but performance is optimized by maximizing the alignment of the antenna and implant and minimizing the initial distance between these two components.

In spite of the standard antenna design’s excellent *in silico* results for the structure displacement sensing applications, this design tends to be rather large (i.e., *length* × *height* = 138 mm × 100 mm, where length = *L* + *L*_*r*_ + 2*R* + *L*_*k*_ and height = 2[*H* + *H*_*d*_], Fig. 1 and Table 1), and exhibits poor benchtop sensitivity to small profile metallic structures (Fig. 7c). The miniaturized antenna, however, features a total calculated *length × height* = 36 mm × 30 mm, which amounts to an approximately 13-fold reduction in total surface area. Despite this area reduction, comparing Fig. 6c to 6d and Fig. 7a to 7b, it can be concluded that miniaturization of the antenna did not degrade its sensitivity performance. In fact, antenna sensitivity was actually improved substantially (i.e., the same variations in plate offsets yield even higher resonant frequency shifts in the same bandwidth). The appreciable reduction in antenna size has the additional benefit of facilitating antenna mounting to a precision linear actuator.

The results of Fig. 8 suggest that resonant frequency changes are irrespective to whether the antenna or metallic structure was displaced to change the relative distance between the two members. Despite the perceptive triviality of this observation, these data represent a gestalt whose clinical importance cannot be overstated. The primary limitation of direct electromagnetic coupling antennas, as an orthopaedic diagnostic tool, is the highly nonlinear relationship between resonant frequency shift and antenna-metallic structure distance (Fig. 6 & Fig. 7). As a clinical tool, controlled mechanical loads are applied to a fractured limb and the resultant deflections of implanted metallic structures (i.e., plates, rods, and screws used to stabilize fractured bone segments) are noninvasively measured via antenna resonant frequency shifts [22], [25], [26]. The magnitude of these shifts is highly sensitive to the initial antenna-implant distance (Fig. 6 & Fig. 7) [25], which cannot be accurately known nor measured in a clinical setting. Comparison of data collected from different points within the healing timeline is thus an arduous task.

The similarity of the two data profiles in Fig. 8 confirms that knowledge of initial antenna-implant distance is unnecessary for accurate prediction of implant deflections, as long as the position of the antenna can be precisely displaced in the same direction of implant deflections. After diagnostic tests are performed (i.e., collecting resonant frequency shift per applied load), the antenna can be displaced known distances, via linear actuator, from the measurement site while assessing the resultant resonant frequency shift (i.e., resonant frequency shifts per known change in antenna-implant distance). The measured resonant frequency shift per change in antenna-implant distance can be used to calibrate the initial diagnostic test data to produce implant deflection per applied load, which is the inverse of fracture bending stiffness. This metric is essential for clinical diagnosis of fracture healing progression; thus, additional studies are warranted to further explore the ability of the proposed technique for accurate prediction of bone fracture bending stiffness.

## V. CONCLUSION

The antennas developed in this study are intended for use in orthopaedic diagnostic applications. These antennas have demonstrated efficacy in remotely detecting relative deflections and/or displacements of metallic plates of alloys similar to those used to treat fractured bones. While these results are promising, it should be noted that clinically available orthopaedic fixation hardware is highly varied in structure/design/material. Regardless, the diagnostic application of this technology relies upon relative and repeated resonant frequency shifts, and thus this technology / approach is applicable to any metallic implant design that is used to mechanically stabilize any body part. However, to fully characterize the extent of clinical applicability of this technology with different implant designs and implementations, additional translational studies should be performed in translational and/or clinical models. Future studies are therefore recommended for evaluating the applicability of the miniaturized antenna for predicting healing induced bone fracture stiffness progression. These results nonetheless agree with previous antenna development studies [25], which were foundational to the development of subsequent clinical diagnostic devices [26], [27]. Previous antenna designs were notably limited by highly nonlinear antenna sensitivity, as a function of initial antenna-implant distance, which made clinical implementation of this technology challenging. The miniaturized antenna developed in this study marks a pronounced improvement of this technology due to its appreciably reduced size while still maintaining excellent antenna sensitivity. This antenna will thus enable the implementation of calibration techniques to mitigate nonlinear antenna effects, and enable direct prediction of essential indicators of bone fracture healing progress (fracture stiffness). Notably, the exclusivity of the proposed miniaturized Vivaldi antennas, being narrow band by nature, is not claimed (i.e., other antenna types can be used as viable sensors). However, resonant patches, for instance, would have approximately 7 times larger surface and would require cumbersome mounting in front of a thin limb due to their broadside radiation patterns. Hence, the validated excellent properties of the small Vivaldi antennas, with their small footprints and thin forward-looking profiles, still make them excellent sensor candidates, especially for the future integration in small arrays to obtain better sensitivity and resolution. The findings of this study will thus serve as the foundation for developing novel orthopaedic diagnostic technologies.

## Figures and Tables

**TABLE 1.** Vivaldi Antenna Parameters for 1.5 GHz Operation.

Parameter	Standard Antenna	Miniaturized Antenna
*h*	0.1 mm	0.1 mm
*H*	20 mm	10 mm
*H*_*d*_	30 mm	5 mm
*L*	100 mm	30 mm
*R*	15 mm	1 mm
*L*_*k*_	6 mm	2 mm
*L*_*r*_	2 mm	2 mm
*a*	35 m^−1^	10 m^−1^
*w*_*1*_	1 mm	1 mm
*w*_*2*_	2.9 mm	2.9 mm
*l*_*1*_	8 mm	0.95 mm
*l*_*2*_	38 mm	6.17 mm
*l*_*3*_	38 mm	8 mm

**TABLE 2.** Vivaldi antenna resonances around 1.5 GHz as a function of increasing antenna-metallic plate distance.

	Standard Antenna		Miniaturized Antenna	
Plate offset (mm)	Resonan *f* (GHz)	|*S*_11_| (dB)	Resonant *f* (GHz)	|*S*_11_| (dB)
0.1	1.20	−7.75	1.21	−18.49
0.5	1.33	−9.80	1.44	−33.44
1	1.36	−10.64	1.58	−27.57
2	1.40	−11.84	1.66	−37.94
3	1.43	−13.06	1.72	−39.23
4	1.44	−14.66	1.74	−33.96
6	1.47	−16.37	1.78	−33.58
8	1.48	−18.65	1.80	−20.28
10	1.49	−20.83	1.82	−22.99
